# A Systematic Review and Meta-Analysis of Validation Studies Performed on Dietary Record Apps

**DOI:** 10.1093/advances/nmab058

**Published:** 2021-05-21

**Authors:** Liangzi Zhang, Andreja Misir, Hendriek Boshuizen, Marga Ocké

**Affiliations:** National Institute for Public Health and the Environment (RIVM), Bilthoven, The Netherlands; Division of Human Nutrition, Wageningen University and Research, Wageningen, The Netherlands; Division of Human Nutrition, Wageningen University and Research, Wageningen, The Netherlands; National Institute for Public Health and the Environment (RIVM), Bilthoven, The Netherlands; Division of Human Nutrition, Wageningen University and Research, Wageningen, The Netherlands; National Institute for Public Health and the Environment (RIVM), Bilthoven, The Netherlands; Division of Human Nutrition, Wageningen University and Research, Wageningen, The Netherlands

**Keywords:** smartphone apps, validation study, review, dietary records, dietary apps, meta-analysis, study design, dietary assessment, mobile technologies, dietary intake

## Abstract

Mobile dietary record apps have been increasingly validated by studies with various study designs. This review aims to evaluate the overall accuracy of dietary record apps in measuring the intake of energy, macro- and micronutrients, and food groups in real-life settings and the designs of validation studies. We systematically searched mobile dietary record validation studies published during the period from 2013 to 2019. We identified 14 studies for the systematic review, of which 11 studies were suitable for meta-analyses on energy intake and 8 studies on macronutrient intake. Mean differences and SDs of nutrient estimations between the app and the reference method from studies were pooled using a random-effects model. All apps underestimated energy intake when compared with their reference methods, with a pooled effect of −202 kcal/d (95% CI: −319, −85 kcal/d); the heterogeneity of studies was 72%. After stratification, studies that used the same food-composition table for both the app and the reference method had a lower level of heterogeneity (0%) and a pooled effect of −57 kcal/d (95% CI: −116, 2 kcal/d). The heterogeneity of studies in the differences in carbohydrate, fat, and protein intake was 54%, 73%, and 80%, with the pooled effect of −18.8 g/d, −12.7 g/d, and −12.2 g/d, respectively, after excluding outliers. The intakes of micronutrients and food groups were statistically nonsignificantly underestimated by the apps in most cases. In conclusion, dietary record apps underestimated food consumption compared with traditional dietary assessment methods. Moreover, varying study designs have been found across studies. Recommended practices for conducting validation studies were formulated including considering biomarkers as the reference, testing in a larger and more representative study population for a longer period, avoiding the learning effect of each method, and comparing food group or food item consumption in addition to comparing energy and nutrient intakes.

## Introduction

Diet has been recognized as one of the determinants for developing noncommunicable diseases such as cardiovascular disease, diabetes, and cancer (1). An accurate assessment of dietary intake is fundamental in understanding diet and health relations (2). Self-reported dietary intake is the most commonly used method in large-scale nutritional studies, which can assess all food and nutrients and has a better trade-off between cost, response, and accuracy than more objective measures (e.g., biomarkers) (3). However, self-reported intake is subject to response error (e.g., inaccurate recall, under- and overreporting) and portion-size error (e.g., inaccurate portion-size estimation) (4, 5). Retrospective methods such as 24-h dietary recalls (24HRs) are dependent on respondents’ memory (5), while in prospective methods such as dietary records, respondents are able to estimate portion sizes in real time with household measures (6), but are more likely to adjust their dietary intakes out of social desirability (7).

Due to the error-prone nature and burdensome procedures in existing dietary assessment methods, technology advancements have favored the use of digital applications in assessing dietary intakes (8–10). We have seen exponential growth in mobile phone ownership in the past 2 decades, providing a convenient platform for recording dietary intake (11). Mobile applications, which are constructed based on the theory of traditional dietary assessment methods, are increasingly applied in nutritional studies (11). Most mobile dietary apps align with dietary records due to the portable nature of smartphones and the ability to incorporate real-time recording features like barcode and image taking (12). Although image-based or image-assisted apps have also been increasingly developed, they are still in the phase where large investments in personnel assistance and in advancing computer algorithms are required (13). Hence, textual food input has been the dominant method in apps for both commercial and research purposes.

Although the underlying method (e.g., dietary record) in most apps is not new, the technology and workflow are new, which changes the method of food input entirely. Therefore, dietary record apps should be validated in estimating dietary intakes before being applied to large-scale research. Validation studies assess the degree to which a new method measures what it is intending to measure by comparing it with a reference method (14). The reference method should have a higher degree of demonstrated validity and have uncorrelated errors with the test method (15). Eldridge and colleagues (8) found that apps developed for research use have been validated more frequently with a well-established dietary assessment method than commercial apps that usually focus on personal dietary tracking.

The quality of existing validation studies depends on the resources and methodologies that researchers can access (8). There are no recent reviews on the results of validation studies that specifically focused on dietary record apps. A review by Sharp et al. (9) focused on evaluating the validity, feasibility, and acceptability of a broader range of technologies, including both dietary apps and image-based technologies validated from 2001 to 2013. They concluded that these technologies showed similar, but not superior, validity when compared with conventional methods. One of the studies that Sharp et al. included, which was published in 2013 by Carter et al. (16), was stated to be the first study on dietary record app validation. It is likely that many new dietary record apps have been developed and validated since this study. Apart from reviewing the new evidence from these validation studies, a meta-analysis on results across different validation studies, along with a critical evaluation of the study designs, could provide more information on the accuracy of using dietary record apps in real-life situations.

Thus, this systematic review aims to evaluate the current state of the overall accuracy of mobile phone dietary apps in estimating the intake of energy, macronutrients, micronutrients, and food groups, using a meta-analysis when applicable. Also, this study aims to review the design and methodology of dietary record app validation studies.

## Methods

The literature search for this study was undertaken from 1 September to 1 November 2019. We searched studies published in English in Web of Sciences and its regional databases including Current Contents Connect, Korean Journal Database, Russian Science Citation Index, and SCIELO Citation Index. Additional searches were performed in PubMed, Medline, and Google Scholar. We also scrutinized citations from already detected studies and review articles. Since the previous review by Sharp et al. (17) covered studies from 2001 to 2013, this study aimed to collect studies from 1 January 2013 to 31 October 2019, including the first validation study on a smartphone dietary record app from Carter et al. (16) published in 2013. The following search strategy was used: (“smartphone” OR “phone” OR “mobile” OR “app” OR “mobile app*”) AND (“diet* record” OR “dietary assessment” OR “food intake” OR “dietary measurement” OR “energy intake” OR “caloric intake” OR “nutrient intake” OR “nutrition assessment” OR “diet tracking” OR “food tracking”) AND (“valid*” OR “accuracy” OR “compar*” OR “evaluat*”) in the abstract, title, or keywords.

### Study identification and data extraction

Studies were potentially eligible for inclusion in this systematic review if they satisfied all of the following criteria: *1*) exclusively self-reported dietary record apps with automatic nutrient estimations, *2*) included a validation that compared the app with an objective method (e.g., biomarker or accelerometer) or with a reference dietary assessment method, *3*) studies with a sample of participants entering all foods and beverages consumed on a day in a community-dwelling situation, and *4*) validation studies covering any segment of the global population and all genders. Two researchers (AM, LZ) performed study screening independently and were blinded to the web application Rayyan (18). After the first screening looking at titles and abstracts, agreement on the list of selected papers was reached between the reviewers. Full articles were then retrieved and were further assessed for eligibility, independently and blinded, by the 2 researchers. The final decision on the inclusion of studies was based on a consensus between the 2 researchers and discussed with their supervisor, if necessary. This systematic review protocol was developed following the Preferred Reporting Items for Systematic Reviews and Meta-Analyses (PRISMA) statement (19).

The features and results of each validation study were extracted consecutively by 2 researchers (AM extracted the data and LZ checked the data for accuracy and vice versa). General characteristics of the validation studies, such as the type of reference method, the choice of a time frame, the sequence and spacing of using the app and the reference methods, the selection and the number of subjects, and the applied statistical tests, were extracted. Mean differences in energy and macronutrient intakes were extracted between the app and the reference method for further meta-analysis. Energy intake was transformed into kilocalories if it was only available in kilojoules. For studies in which multiple days were compared, only the average of the total period or only data where the number of participants satisfied the power calculation for studies were taken into account [e.g., Chen et al. (20)]. The correlation coefficients (Pearson's *r* and Spearman's ρ) and limits of agreement (LOAs) were collected where available. The correlation coefficients were categorized based on Chan (21) and Akoglu (22) into strong if *r* ≥ 0.80, moderate if 0.60 ≤ *r* < 0.80, fair if 0.30 ≤ *r* < 0.60, and poor if *r* < 0.30. For studies where other nutrients and food groups were measured, correlation coefficients and under- or overreporting between the app and the reference methods are presented.

### Meta-analysis

The meta-analysis of energy and macronutrients was performed on studies that had enough uniformity of available data for the dietary component under analysis. Studies were included for meta-analysis if they presented a mean and SD for the app and the reference method (so-called raw effect size data that were most consistent between reviewed studies), and their units for macronutrients were in grams. Pooled mean differences (and 95% CIs) between the app and the reference method were calculated using the Hartung-Knapp-Sidik-Jonkman (HKSJ) random-effect model. The HKSJ model has fewer false positives with a small number of studies than the more common DerSimonian-Laird estimator (23). The Sidik-Jonkman estimator for τ^2^ (and 95% prediction intervals) was used for estimating the variance of the distribution of true effect sizes. Chi-square test (24) at the significance level of *P* < 0.05 was performed with the *I*^2^ statistic, in which cutoffs between 25% to 50%, 50% to 75%, and >75% indicate low, moderate, and high heterogeneity, respectively (25).

When the test showed significant heterogeneity, the sources of heterogeneity were explored with a stratification analysis by 2 characteristics of the validation study (i.e., the reference method used in the study and whether the same food-composition table was used in the app and the reference method). Stratification was performed only on the validation of dietary components if the number of validation studies was ≥10.

Sensitivity analyses were conducted to examine the impact of outlier studies. The outliers were identified: first, if the individual study's CI did not overlap with the CI of the pooled effect; second, the Graphic Display of Heterogeneity (Gosh) plot method was used to detect potential outliers, in case there were studies with CIs that only slightly overlapped with pooled CIs (26). The test could detect studies that might potentially contribute to the heterogeneity. Sensitivity analysis was performed for the intake of both energy and macronutrients by omitting the outlier study.

In the case of ≥10 contributing studies, the potential for publication bias was analyzed with Egger's test (27). Data were analyzed with the statistical program R-Studio® version 1.2.5019, R® version 3.6.1; R packages used include meta, metaphor, esc, and dmetar (R Foundation for Statistical Computing).

## Results

**FIGURE 1:**
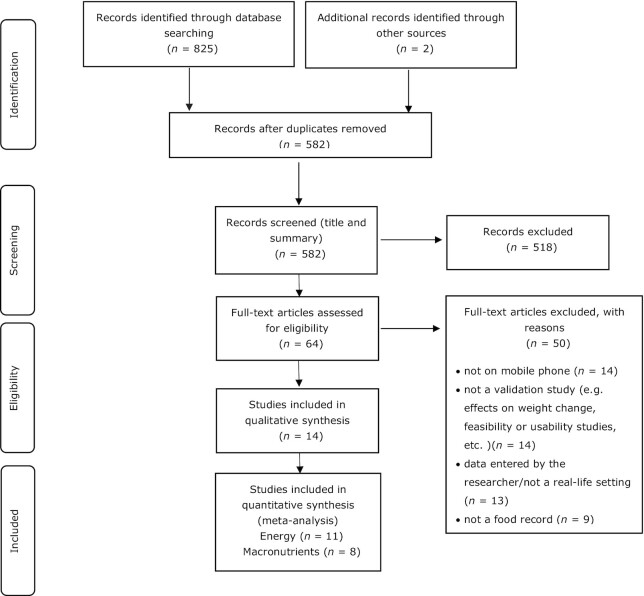
PRISMA flow diagram indicating the number of articles included at each phase. PRISMA, Preferred Reporting Items for Systematic Reviews and Meta-Analyses.

The database searches yielded 825 publications when search results were combined, and 2 additional articles were identified through other sources (search alerts in searched databases). After duplicate records were removed, the title and abstract of 582 studies were screened, which resulted in the exclusion of 518 studies. Because our study focused on validation studies of dietary record apps, studies were excluded if they evaluated weight changes before and after app use, investigated the feasibility or usability of apps, or did pilot testing of apps. After applying inclusion and exclusion criteria, 14 studies were selected for the systematic review, of which 11 studies were selected for meta-analysis on energy intake and 8 studies were selected for meta-analysis on macronutrient intake (see **Figure 1**).

**Table 1** shows the app characteristics and design aspects of each validation study. The 14 studies focused on 12 different apps, of which 7 provided feedback on nutrient intake (16, 20, 28–34) and 5 others did not (12, 35–38). Most validation studies included young adults as their sample population or advertised in a university setting, while 2 studies explicitly mentioned including a wider age range of participants (29, 37). Most validation studies had a medium to small sample size (from 18 to 81 participants), while 2 studies had a larger sample size of 362 and 189 participants (29, 35). The period of app use ranged mostly from 2 to 7 d and contained at least 1 weekend day for most studies, while 2 studies asked participants to record every day for 3 mo (28, 29). The app use was on nonconsecutive days for 3 of the studies (12, 30, 36). Ten studies used 24HRs as the only reference method for 2 d (*n* = 6) (16, 20, 28, 34, 37, 38) or 3 d (*n* = 4) (32, 33, 35, 36). One study used an FFQ (29), 1 study used dietary records (30), 2 studies used an accelerometer (to measure energy expenditure) (12, 31), and 1 study used a combination of accelerometer, 24HRs, and dietary records (34). Among studies with different days of using the app and the reference method, most studies compared the mean of each method averaged across all corresponding days (28, 32–34, 38). Apart from 2 studies using accelerometers exclusively (12, 31), 3 studies used different food-composition databases (FCDs) for the app and the reference method (20, 30, 35), while 2 studies did not specify the FCD used for each method (28, 29).

**TABLE 1:** General characteristics of the 14 dietary record apps and their validation studies^1^

First author, year (ref)	App	Country	Sample size, *n*	Population group, age, y	App days	Reference method days	Feedback on nutrients?	Same FCD in 2 methods?
Lee, 2017 (28)	Diet-A	Korea	21	High school students, 16–18	Every day in 3 mo	Two 24HRs, 1 before app use, 1 after app day	Yes (immediate)	Unspecified
Chen, 2019 (20)	MyFitnessPal (MFP)^2^	Australia	45^3^	University students and staff; mean: 32	4 consecutive days (including weekend)	Two 24HRs, unannounced, 1 d after each app day	Yes (immediate)	No
Recio-Rodriguez, 2019 (29)	EVIDENT II	Spain	362^3^	Adult, 18–70	Every day in 3 mo	One FFQ, before 3-mo app use	Yes (end of the day)	Unspecified
Wellard-Cole, 2019 (35)	Eat and Track (EaT)	Australia	189	Young adults, 18–30	3 consecutive days, starting days staggered across the population (some include weekend)	Three 24HRs, 1 d after each app day	No	No
Teixeira, 2018 (30)	MyFitnessPal (MFP)^2^	Brazil	30	University students, 18–30	2 nonconsecutive days (at the end of a weekday and a weekend)	Food record (paper), at consumption	Yes (immediate)	No
Pendergast, 2017 (12)	FoodNow	Australia	56	Young adults, 18–30	4 nonconsecutive days (1 weekend)	Accelerometer, 7 d with app day	No	—
Svensson, 2015 (31)	—	Sweden	81^3^	Adolescents, 14–16	3 consecutive days (some include weekend)	Accelerometer, 3 d with app day	Yes (after each record submission)	—
Mescoloto, 2017 (36)	Nutrabem^2^	Brazil	40^3^	University students; mean: 21	3 nonconsecutive days (1 weekend); 2-wk interval in-between	Three 24HRs, 1 d after each app day	No	Yes
Bucher Della Torre, 2017 (37)	e-CA	Switzerland	18	Adults, 20–60	5 consecutive days including at least 1 weekend	Two 24HRs, unannounced, 1 d after each app day	No	Yes
Ambrosini, 2018 (38)	Research Food Diary (RFD)^2^	Australia	50	University students and staff; mean: 31	4 consecutive days (including weekend)	Two 24HRs, 1 after an app day, 1 on weekend within 7 d of app day	No	Yes
Carter, 2013 (16)	My Meal Mate (MMM)^2^	UK	41	University students and staff; mean: 35	7 consecutive days (including weekend)	Two 24HRs, unannounced, 1 d after each app day	Yes (immediate)	Yes
Rangan, 2015 (32)	e-DIA	Australia	80	University students, 19–24	5 consecutive days (3 week days, 2 weekend days)	Three 24HRs, unannounced, 1 d after each app day	No	Yes
Rangan, 2016 (33)	e-DIA	Australia	80	University students, 19–24	5 consecutive days (3 week days, 2 weekend days)	Three 24HRs, unannounced, 1 d after each app day	No	Yes
Lozano-Lozano, 2018 (34)	BENECA	Spain	20^3^	Breast cancer survivors; mean; 47.5	6 consecutive days (including weekend)	Accelerometer, 8 d with app day 2 24HR, unannounced, 1 d after each app day 4 DRs, with app day, unclear sequence	Yes (immediate)	Yes

1 DR, dietary record; FCD, food-composition database; FFQ, food-frequency questionnaire; ref, reference; 24HR, 24-h recall.

2 App that can be downloaded from Apple/Google store.

3 Power analysis was done.

**Table 2** shows the included nutrients and statistical tests in each study. Ten studies investigated the energy and macronutrient intake, while 6 of them also compared micronutrient intake (28, 29, 32, 35, 36, 38). Four studies looked at food group intakes (33, 34, 36, 37). In terms of statistical parameters and tests, the frequency of using a paired *t* test was the highest (*n* = 12), followed by correlation coefficients (*n* = 11) and Bland-Altman LOAs (*n* = 11). Thirteen studies used at least 2 statistical parameters, 8 studies used all 3 parameters, while Lee et al. (28) only used the *t* test.

**TABLE 2:** Nutrients and statistical tests included in the validation studies of 14 dietary record apps^1^

First author, year (ref)	App	Energy	Macronutrients^2^	Micronutrients	Food groups	Significance test	Correlation	LOAs
Lee, 2017 (28)	Diet-A	√	5	3		√		
Chen, 2019 (20)	MyFitnessPal (MFP)	√	4			√	√	√
Recio-Rodriguez, 2019 (29)	EVIDENT II	√	9	20		√	√	√
Wellard-Cole, 2019 (35)	Eat and Track (EaT)	√	6	1		√	√	√
Teixeira, 2018 (30)	MyFitnessPal (MFP)	√	5			√	√	√
Pendergast, 2017 (12)	FoodNow	√					√	√
Svensson, 2015 (31)	No name	√				√	√	√
Mescoloto, 2017 (36)	Nutrabem	√	4	3	12	√	√	
Bucher Della Torre, 2017 (37)	e-CA	√	4		2	√		√
Ambrosini, 2018 (38)	Research Food Diary (RFD)	√	8	2			√	√
Carter, 2013 (16)	My Meal Mate (MMM)	√	4			√	√	√
Rangan, 2015 (32)	e-DIA	√	10	14		√	√	√
Rangan, 2016 (33)	e-DIA				8	√	√	√
Lozano-Lozano, 2018 (34)	BENECA		1		1	√		

1 LOA, limit of agreement; ref, reference.

2 Includes subgroups of macronutrients, such as saturated fat, fiber, sugar, etc.

**FIGURE 2:**
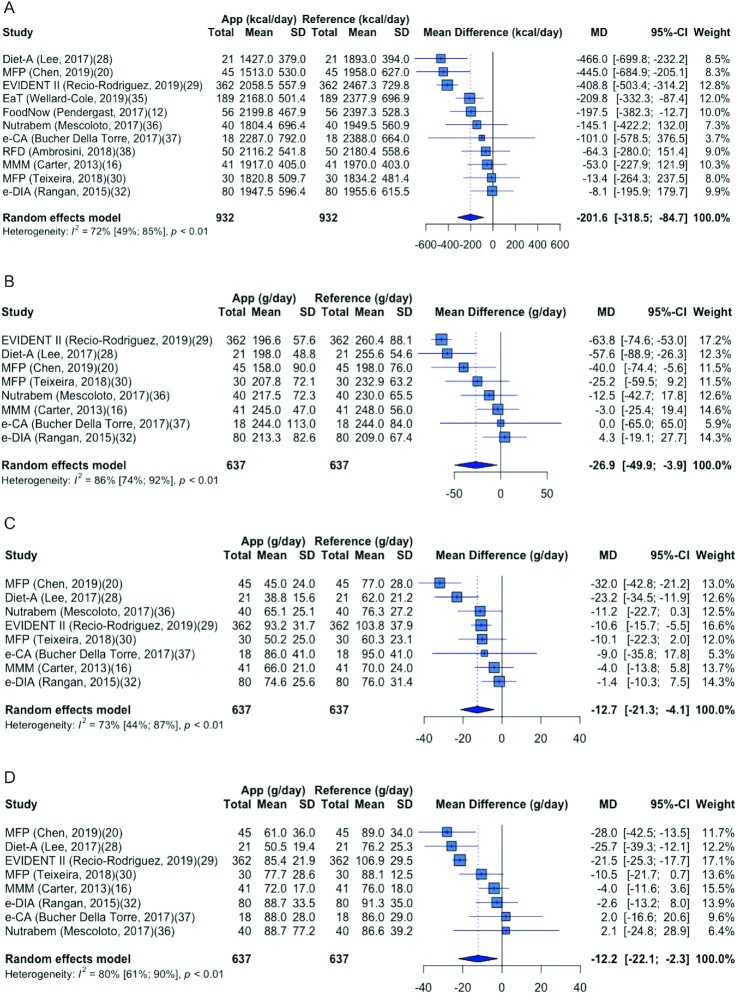
Forest plot for the mean difference in energy and macronutrient intake between the app and the reference method in included validation studies. (A) Energy, (B) carbohydrate, (C) fat, and (D) protein. EaT, Eat and Track; MFP, MyFitnessPal; MMM, My Meal Mate; RFD, Research Food Diary.

Meta-analysis was performed on 11 studies for energy intake and 8 studies for macronutrient intake. **Figure 2**A shows the pooling of the mean difference in energy. All apps underreported mean energy intake when compared with the reference method, with a pooled effect of −202 kcal/d (95% CI: −319, −85 kcal/d). The heterogeneity of studies expressed as *I*^2^ was 72%, which fell into the upper-moderate to high heterogeneity group. Stratification was first performed between the 8 studies that used 24HRs as a reference method and the 3 studies that used all “other” reference methods. In the 24HR group, a lowered pooled mean difference of −186 kcal/d (95% CI: −334, −37 kcal/d) was found, and heterogeneity of studies dropped to 59%. Then, stratification was performed on 12 studies that either used “the same” or “different” FCDs for the app and the reference method. The pooled mean difference in the group of studies with the same FCD decreased to −57 kcal (95% CI: −116, 2 kcal/d), and the heterogeneity of studies dropped to 0%. Heterogeneity was also explored with sensitivity analysis to exclude outlying studies. No outliers were detected by looking at the overlapping of CIs of each study with the pooled effect. Using the Gosh plots method, the EVIDENT II app (29) was detected as an outlier. The pooled effect dropped to −171 kcal/d (95% CI: −288, −54 kcal/d), and the heterogeneity of studies dropped to *I*^2^ = 52% after deleting the outlier. Egger's test (*P* = 0.17) indicated no evidence of study bias.

Pooling of the effect sizes on carbohydrate, fat, and protein intake was performed in 8 studies (see Figure 2B–D). The pooled effects were negative for all 3 macronutrients. High heterogeneity was found in studies in estimating both carbohydrate (*I*^2^ = 86%) and protein (*I*^2^ = 80%), with a pooled effect of −26.9 g/d and −12.2 g/d, respectively. Similar to energy, the EVIDENT II app was detected as an outlier for carbohydrate comparisons (29). After deleting the data on the outlier, the heterogeneity dropped to moderate (*I*^2^ = 54%), with a pooled effect of −18.8 g/d. The heterogeneity of studies comparing fat intake was slightly lower than carbohydrate and protein (*I*^2^ = 73%), with a pooled effect of −12.7 g/d. In all 8 studies, the app underreported mean fat intake when compared with the reference method.

When looking at the performance of each app, e-DIA had a relatively lower mean difference and variance in the intake of energy and all macronutrients than other apps (32). The app e-CA had the lowest mean difference for both carbohydrate and protein (37). However, the SD of the differences was the highest among all studies for energy, carbohydrate, and fat. Diet-A (28) and MyFitnessPal (MFP) [from Chen et al. (20)] had the highest mean difference across energy, fat, and protein.

**Table 3** illustrates the correlation coefficient and LOAs between the apps and the reference methods for the intake of energy and macronutrients. The column with LOAs represents the distance between the upper and the lower limit. Five studies reported both correlation and LOAs for energy and all macronutrients. For energy, 3 studies that had a weak correlation between the 2 methods had larger LOAs than other studies (20, 29, 31). Most studies had a moderate correlation, with a range of 0.60 to 0.80. The distances of LOAs were mostly within 2000 kcal, with 1 exception of 2223 kcal. Nutrabem had the highest correlation for energy, carbohydrate, and protein (36). My Meal Mate (MMM) had the highest correlation in fat (16). The app e-Dia had similar correlations for energy and all macronutrients, from 0.64 to 0.79 (32). EVIDENT II had weak correlations for all macronutrients and energy (29). The average correlation across studies was 0.54 to 0.60; energy and fat intakes were both the lowest at 0.54. The average across energy and macronutrients in each study ranged from 0.23 to 0.78, with the majority of the studies in the moderate category. The expression of macronutrient intake differed between studies, with grams, energy percentages, and natural logarithms.

**TABLE 3:** Summary of the correlation coefficients and LOAs for energy and macronutrient comparisons between apps and reference methods^1^

			Energy		Carbohydrates		Fat		Protein		
First author, year (ref)	App	*n*	*R*	LOAs, kcal/d	*r*	LOAs, g/d	*r*	LOAs, g/d	*r*	LOAs, g/d	Mean *r* (energy and macronutrients)
Chen, 2019 (20)	MyFitnessPal (MFP)	45	0.29	2727	0.41	357	0.16	131	0.43	136	0.32
Recio-Rodriguez, 2019 (29)	EVIDENT II	362	0.23	3263	0.27	—	0.23	—	0.20	—	0.23
Wellard-Cole, 2019 (35)	Eat and Track (EaT)	189	0.67	2223	0.79	21%	0.56	25%	0.73	14%	0.69
Teixeira, 2018 (30)	MyFitnessPal (MFP)	30	0.67	1345	0.41	123^2^	0.58	67^2^	0.43	32	0.52
Pendergast, 2017 (12)	FoodNow	56	0.75	1383	—	—	—	—	—	—	—
Svensson, 2015 (31)	No name	81	0.13	2639	—	—	—	—	—	—	—
Mescoloto, 2017 (36)	Nutrabem	40	0.77	—	0.82	—	0.71	—	0.83	—	0.78
Bucher Della Torre, 2017 (37)	e-CA	18	—	447	—	266	—	104	—	75	—
Ambrosini, 2018 (38)	Research Food Diary (RFD)	50	0.52	2126	0.72	23%	0.63	22%	0.79	12%	0.67
Carter, 2013 (16)	My Meal Mate (MMM)	41	0.68	1065	0.57	—	0.75	—	0.57	—	0.64
Rangan, 2015 (32)	e-DIA	80	0.66	1965	0.64	274	0.68	92	0.79	88	0.69
Average			0.54	1918	0.58		0.54		0.60		

1 LOA, limit of agreement; ref, reference.

2 Back-transformed value.

**Table 4** lists other nutrients that were most commonly assessed in the included studies. In most studies, the app underestimated nutrient intakes. Calcium and sodium intake in Diet-A and fiber and alcohol in EVIDENT II were statistically significantly underestimated, while the rest of the underestimated nutrients were all nonsignificant. Alcohol intake was significantly overestimated in Research Food Diary (RFD). Rangan et al. (32) compared all nutrients in this table and had the second-highest average correlation among the nutrients, while EVIDENT II had the lowest average correlation across most nutrients, except for alcohol. Eat and Track (EaT) had the highest average correlation among the included nutrients, mainly due to the strong correlation for sugar intake.

**TABLE 4:** Summary of the under-/overestimation of apps and correlation coefficients with the reference methods for macronutrient subgroups and micronutrients

First author, year, reference	App	Compared with reference	Calcium	Iron	Sodium	Vitamin C	Saturated fat	Sugar	Fiber	Alcohol	Mean *r*
Lee, 2017 (28)	Diet-A	Under- or overreporting	Under^1^	Under	Under^1^	—	Under	—	—	—	—
		*r*	—	—	—	—	—	—	—	—	—
Recio-Rodriguez, 2019 (29)	EVIDENT II	Under- or overreporting	—	—	—	—	Under	—	Under^1^	Under^1^	—
		*r*	0.32	0.26	0.15	0.32	0.31	—	0.31	0.68	0.34
Wellard-Cole, 2019 (35)	Eat and Track (EaT)	Under- or overreporting	—	—	Under	—	Under	Under	—	—	—
		*r*	—	—	0.56	—	0.59	0.82	—	—	0.66
Mescoloto, 2017 (36)	Nutrabem	Under- or overreporting	Under	Under	—	Under	—	—	—	—	—
		*r*	0.57	0.66	—	0.6	—	—	—	—	0.61
Ambrosini, 2018 (38)	Research Food Diary (RFD)	Under- or overreporting	Over	Over	—	—	Under	Under	Under	Over^1^	—
		*r*	0.45	0.42	—	—	0.60	0.68	0.66	0.65	0.58
Rangan, 2015 (32)	e-DIA	Under- or overreporting	Under	Over	Under	Under	Under	Under	Over	Over	—
		*r*	0.75	0.57	0.60	0.68	0.75	0.56	0.54	0.77	0.65

1 Statistically significant estimation, *P* < 0.05.

Food groups were only validated for 4 apps (e-CA, Nutrabem, BENECA, e-DIA). A different categorization of food groups was found across studies; differences in dairy, fruits, vegetables, meat, and grain intake were most commonly reported. Food group intakes were underestimated by apps, but this was mostly not statistically significant. In the BENECA app, vegetables and fruits were mostly underreported by participants. Among studies that investigated correlations, the highest correlation found for the Nutrabem app was poultry (*r* = 0.85) and lowest was for nuts (*r* = 0.31) and vegetable oils (*r* = 0.37). The app e-DIA had relatively stronger correlations among all included food groups, from 0.75 to 0.88, and had an equal number of under- and overestimations.

## Discussion

This paper aimed to evaluate the validity of dietary intake assessed with mobile phone dietary record apps. More than half of the apps from 14 included studies were validated in university settings, were of a small scale with a duration of 2 to 7 consecutive days, used 24HRs as the reference method, and used the same FCDs for the test and the reference method. The meta-analysis on energy and macronutrient comparisons found that dietary record apps underreported energy and macronutrients relative to more traditional dietary assessment methods. Moderate heterogeneity was reached when an outlier study was excluded from the meta-analysis for energy and carbohydrate. Studies using the same FCD for the apps and the reference methods had no heterogeneity for energy intake and had a lowered pooled effect of −57 kcal. Studies that observed smaller differences in energy intake between the app and the reference method also had smaller differences in macro- and/or micronutrients and food groups.

### Intentional/unintentional underreporting

Underreporting of energy intake in the app compared with the reference method was found in all studies. An even larger extent of underreporting was expected for studies because the reference method that most studies used is also subject to underreporting when compared with recovery biomarkers (8). The tendency to underreport when using the app or other self-report methods may either be unintentional and/or intentional (11). The effect of unintentional underreporting could potentially be alleviated by adding adequate prompts and improving technological add-ins (38), whereas intentional underreporting is more challenging to eliminate when participants deliberately omit the input of certain foods out of social acceptability or convenience (39). In the current study, a larger extent of underestimation in carbohydrate and fat intake was found as compared with protein, which is in line with the findings from another review on a technology-based dietary assessment tool by Eldridge et al. (8). As Bucher Della Torre et al. (37) and Chen et al. (20) specified, people forget to report fat, alcohol, discretionary food and beverage (high in fat/sugar) intakes easily unless prompted by interviewers, while Rangan et al. (32) indicated that people intentionally underreport added sugar and alcohol while using the app.

Approximately half of the errors in energy intake estimations from dietary records administered on technological devices have been attributed to wrong portion-size estimations (40). Participants were asked to refer to a provided food model booklet to assist with the estimation of portion sizes during 24HRs, while most apps provide metric weights (e.g., grams, milliliters) or household measure options (e.g., cups) with no portion-size images (41). Bucher Della Torre et al. (37) found that participants tended to choose the app-proposed portions even if their real portions were different, especially with drinks. Mobile technologies with the assistance of digital photographs have shown a lesser extent of underestimation than regular dietary records in a community-dwelling situation compared with doubly labeled water (DLW) (42–44). These studies were not included in the current review because participants did not exclusively self-report and required a great amount of involvement of dietitians to identify foods and amounts from photos. Automatic food recognition and volume estimation could potentially outperform portion sizes estimated by individuals, but validation is needed to verify the applicability in large-scale studies (45).

Some studies conducted the 24HR the day after using the app, which might have caused a memory effect and reduced the extent of underreporting in the 24HR (16). Moreover, the availability of nutrient feedback and dietary advice from apps could affect the 24HRs performed afterward (46, 47). The learning effect of the 2 methods could be reduced if the app and the reference method are used on separate days (48), or if the records from dietary record apps are deleted before using the reference method. Moreover, conducting the 24HR unannounced was found to help avoid behavioral change (49). A good study design in the arrangement of the methods was found in the study by Ambrosini et al. (38). They conducted the second 24HR unannounced on a different day within 7 d of app use (38). In this way, both the app and reference method are measuring dietary intake to a similar extent while limiting the possible influence of each method.

### Explanations of high heterogeneity

We observed a higher mean difference in studies where different FCDs were incorporated into the app and the reference method. Nutrient discrepancies between different FCDs were also found in studies where the same food items were entered by researchers into apps with different FCDs (8, 50–54). Thus, the effect of “human components” on nutrient estimations, which were mainly accounted for in validation studies, should be distinguished from the use of different FCDs. If it is unfeasible to apply the same FCD in each method, comparing differences in the consumption of food groups or food items between 2 methods could distinguish the source of nutrient discrepancies. In addition, advocacy to move from nutrient-focused research towards food-based research in nutrition epidemiology has stressed the importance of food group validation using new methods (55). Unfortunately, only 4 of the included studies validated food groups, and none of the studies that used different FCDs considered comparing food groups. Studies that compared food group consumption applied different food categorizations and statistical tests, which limited the comparisons of food group differences across studies.

Our results indicate that the choice of the reference method was also one of the determining factors for heterogeneity. The absolute validity was not reported in smartphone application validations, possibly due to the high cost associated with recovery biomarkers and the availability of limited nutrients. When investigating the relative validity of a method it is desirable to use a reference method with uncorrelated errors and better accuracy—for example, comparing dietary records with 24HRs. One included study used an FFQ as a reference before the 3-mo app-use period (29). The FFQ covered the food consumption for the whole year before the app use, which might show a higher variation in food consumption due to factors such as seasonality. In addition, FFQs generally have a lower level of accuracy than 24HRs or dietary records and a limited frequency of consumption options and food lists (56). Conversely, Teixeira et al. (30) tested their app with a paper-based dietary record measuring the food consumption during the same period. Here an overestimation of correlation was expected because the 2 methods share the same embedded errors. Two studies used an accelerometer to assess energy expenditure, which is an objective measure that is less burdensome than DLW (12). However, accelerators have shown over- and underestimation of energy expenditure with different levels of physical activity (57).

Most studies used a diverse range of statistical techniques to facilitate a balanced interpretation of results (32). Correlation coefficients indicate the ability of the app to rank individuals and the strength of the association. Bland-Altman plots reveal the presence, direction, and extent of bias at the group level and the extent of measurement error at the individual level (58). A wide LOA found in most studies was expected because the reference measure itself might have potential errors and does not reflect true intakes (16). The other reason for the high nutrient variation between methods was because only a few days of food consumption were collected for most studies. On the other hand, the majority of studies in this review did not adjust the nutrient intake for energy; only studies with raw data were compared. Rangan et al. (32) found a smaller difference and a higher correlation with values adjusted for within-person variation. Tabacchi et al. (59) also found that the heterogeneity of FFQ validation studies decreased if de-attenuated/energy-adjusted values were used. Hence, presenting nutrient comparisons, with raw values and values adjusted for both energy and within-person variation, helps obtain fewer variations in methods and between studies (60).

The limited number of studies that investigated and compared micronutrient intake indicated that it is still premature to obtain insights into the validity of micronutrient intake using apps. With regard to participant selection, young adults could be a good starting point for app validations since people in this age group were found to have a higher acceptability of using apps and could provide more reliable data (61). However, the absence of other population groups limits the generalizability of the validation results.

### Strength and limitations

The work reported here represents the first known meta-analysis of validation studies of dietary record apps conducted among community-dwelling participants. The analysis provides a detailed comparison of the study design and includes results of micronutrients and food groups. For this study, a systematic search strategy was adopted in searching for eligible papers, and we did not find evidence of publication bias among the included studies. The exclusion of image-based and other technology-based dietary methods enabled us to focus on dietary apps where diets were exclusively self-reported in a real-life setting without the interference of study staff in data entry. The narrowed study selection criteria allowed for easier comparison between studies and more confidence in summarizing factors affecting study results. Still, the small number of studies might have lowered the power of the meta-analysis and limited the investigation of certain analyses (59)—for example, testing for publication bias and exploring heterogeneity with stratification was only possible for energy intake.

### Conclusions

This study focused on the validation of dietary record apps where diets were exclusively self-reported in a real-life setting. The pooled results from the included validation studies showed that using dietary record apps could underestimate energy and macronutrient intakes compared with traditional methods. No specific conclusions could be made on micronutrient and food group comparisons due to limited and noncomparable data. Future studies on evaluating new dietary methods are encouraged to carefully consider the design aspects of a validation study. Strategies such as applying recovery biomarkers as a reference could provide a more accurate estimation of dietary under- or overestimation. Comparing discrepancies in the consumption of food groups or food items between methods could help in specifying the source of the measurement error, especially if 2 methods have different embedded food-composition tables.

## Notes

The authors reported no funding received for this work. Author disclosures: The authors report no conflicts of interest.

Abbreviations used: DLW, doubly labeled water; EaT, Eat and Track; FCD, food-composition database; Gosh, Graphic Display of Heterogeneity; HKSJ, Hartung-Knapp-Sidik-Jonkman; LOA, limit of agreement; MFP, MyFitnessPal; MMM, My Meal Mate; RFD, Research Food Diary; 24HR, 24-h recall.
